# Protocol for investigating light/dark locomotion in larval stage zebrafish using a standardized behavioral assay

**DOI:** 10.1016/j.xpro.2024.103346

**Published:** 2024-10-10

**Authors:** Courtney Hillman, James Kearn, Matthew O. Parker

**Affiliations:** 1 Surrey Sleep Research Centre, University of Surrey, GU2 7XH Guildford, UK; 2 Defence Science and Technology Laboratory (DSTL), Porton Down, SP4 0JQ Salisbury, UK

**Keywords:** High Throughput Screening, Model Organisms, Neuroscience, Behavior

## Abstract

Zebrafish (*Danio rerio*) larvae offer a unique avenue for high-throughput *in vivo* investigation. The light/dark locomotor assay is widely used but lacks experimental consistency. Here, we present a protocol for a standardized light/dark assay by describing the steps for plating, acclimatizing larvae, performing the assay, and preparing drug exposure solutions. We also detail procedures for substance exposure and data analysis.

For complete details on the use and execution of this protocol, please refer to Hillman et al.^1^

## Highlights

• Steps for plating larvae for randomized selection and automated system acclimatization
• Guidance on recording pre-exposure locomotion and post-exposure behavior
• Instructions for preparing chemical solutions and exposing larvae
• Procedures for analyzing behavioral data with the custom R code

## Abstract (editor highlights)

Publisher’s note: Undertaking any experimental protocol requires adherence to local institutional guidelines for laboratory safety and ethics.

## Graphical abstract

**Figure:**
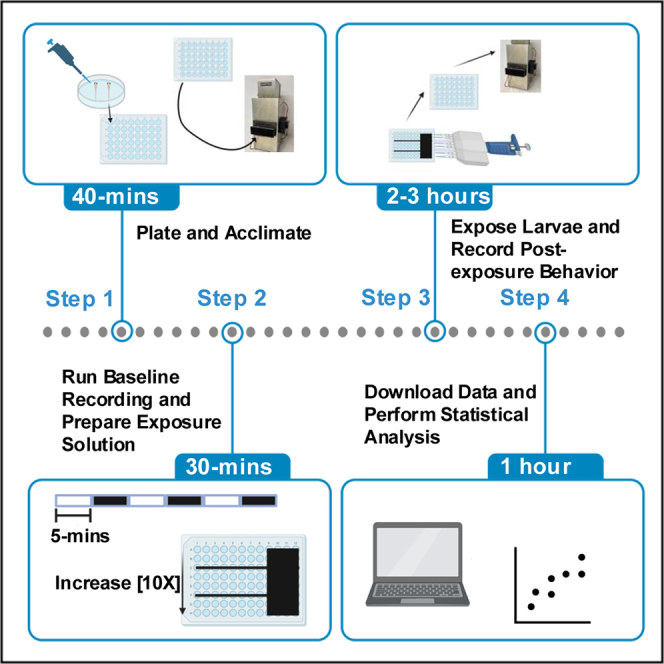

## Before you begin

This protocol will be a step-by-step guide for successful completion of the standardized light/dark behavioral assay in 4 dpf zebrafish larvae using Zantiks MWP automated tracking software (Zantiks, Cambridge, UK [www.zantiks.com]). Here we describe methods for exposing larvae to pharmaceutical substances and recording the behavioral effects. However, this assay is also suitable for non-exposed recordings and determination of genomic phenotypes (see Hillman et al.^1^).

This protocol includes:

 Rearing and raising larvae prior to behavioral investigation.

 Plating, acclimating and recording the baseline larval response in the Zantiks MWP unit.

 Preparing drug solutions and exposure steps.

 Code for tracking and steps for data handling for analysis.

### Institutional permissions

In most countries, the use of animals in experiments must be performed in accordance and with permission from the local Institutional Animal Care and Use Committee (IACUC)/Animal Welfare and Ethical Review Body (AWERB).

### Breeding and rearing of zebrafish

**Timing: 4 days**

This section describes the method for breeding and rearing the zebrafish larvae in preparation for the light/dark behavioral assay.

1. 5 days prior to the larval behavioral assay, prepare adult zebrafish aged between 6–12 months post-fertilization for breeding.
  ***Note:*** Breeding is performed through the addition of substrate (typically marbles) to the home tank and/or pair breeding (see steps 1a and 1b, respectively).
  a. For substrate addition, the night before embryo collection (∼17:00), place a container of substrate into the tank of adult fish (Figures 1A and 1B). The following day, at 1100 - mid-day, remove the container and collect the embryos.

**Figure 1:**
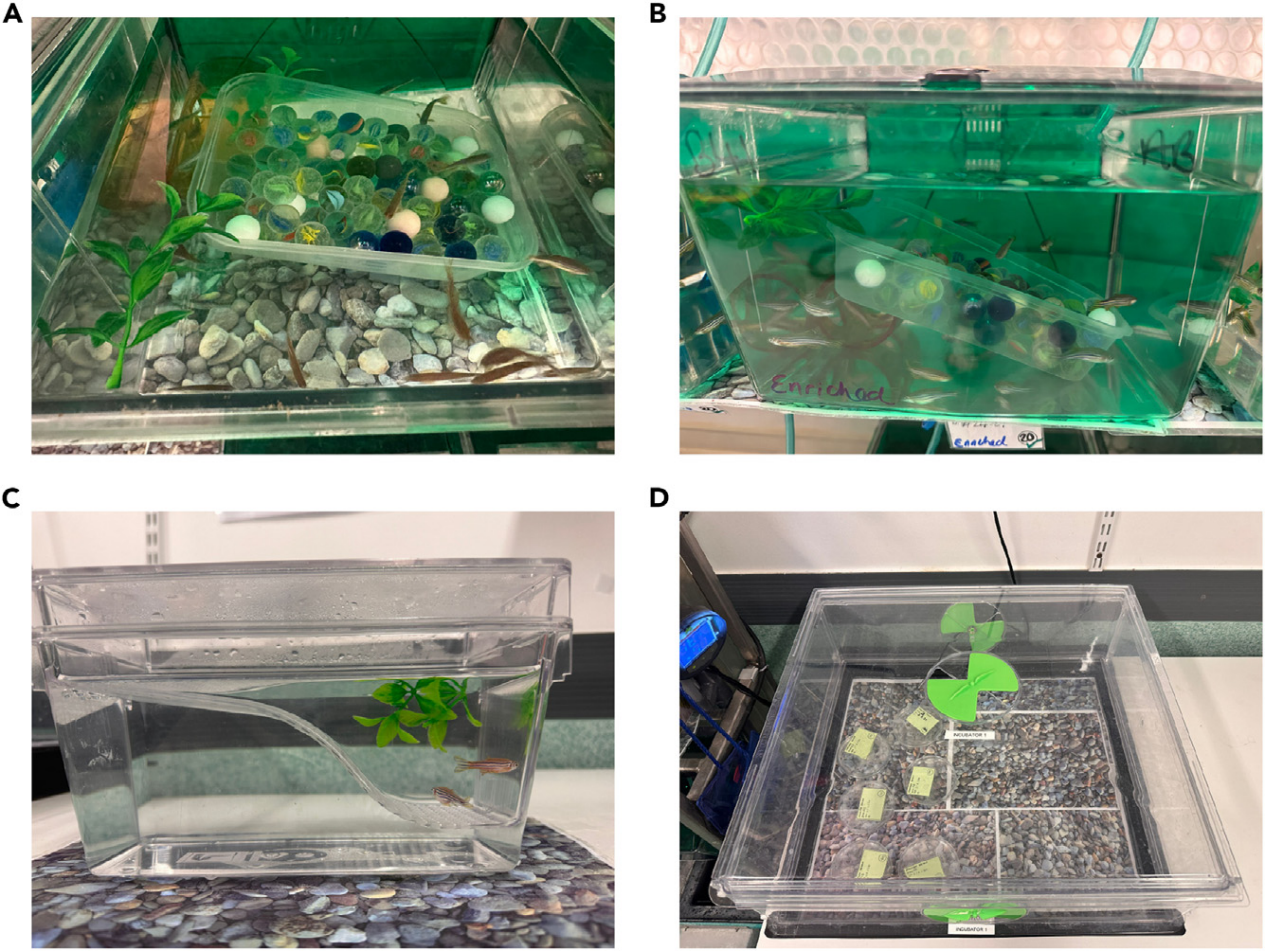
The breeding and rearing set-ups for generating the embryos for the experiment (A and B) show the substrate breeding from two different angles to observe the concept of creating a ‘shallow’ point. (C) is an example of pair-breeding and (D) is the rearing incubator the larvae are placed in following breeding.
  b. For pair breeding, select either one male and one female, or two females and one male, or two males and one female and place into a small tank with a middle divider and a slope. Split the sexes the night before (∼17:00) and the next morning remove the divider when the lights in the facility turn on (∼8:30) and allow 30-min for breeding before returning the adult fish to their home tanks and collecting embryos (Figure 1C).
    ***Note:*** We recommend keeping enrichment (e.g., pebble sheets and plants) in all tanks and incubators (see Figure 1 for reference).
2. Transfer ∼80 0 h-post fertilization (hpf) embryos into labeled 250 mL Petri dishes by pouring the embryos and system water (or the water you use in your lab) and place the dishes into a temperature-controlled incubator (28°C) with a standard 14 h: 10 h light/dark cycle (Figure 1D).

**CRITICAL:** Each morning following breeding perform a complete water change and remove any dead/unfertilized larvae.

### Uploading tracking script to behavioral unit

**Timing: 5 min**

This section describes the setup and use of the Zantiks automated tracking script (available at OSF) for running the light/dark assay using MWP behavioral units.

**CRITICAL:** This protocol is fully adaptable to all automated tracking systems. Ensure that the system is set up to record locomotion as movement in mm/s with 5-min light phase (350 lx, white light) to 5-min dark phase, repeated three times for a total of 30-min.

3. Download the tracking script from the Open Science Framework (OSF) linked to Hillman et al.^1^
4. Connect to the Zantiks Wi-Fi specific for your unit(s).
5. Open the ZANSCRIPT section and upload the script.

***Note:*** All instructions for use of Zantiks is available on their website: www.zantiks.com.

## Key resources table

**Table:** 

REAGENT or RESOURCE	SOURCE	IDENTIFIER
**Software and algorithms**		

Custom R tracking script	This paper	https://osf.io/k7y2c/
RStudio	Posit software	https://posit.co/download/rstudio-desktop/
ZANSCRIPT	This paper	https://osf.io/k7y2c/
Zantiks MWP	Zantiks	https://zantiks.com/products/zantiks-mwp

**Other**		

250 mL Petri dish	Sigma-Aldrich	P5981
48-well plates	Corning Costar	Corning 3548
8 channel multi-channel pipette	Fisherbrand Elite	11835772
96-well plates	Corning Costar	Corning 3548
Aqua-Sed	Vertark	https://www.zmsystems.co.uk/new-aqua-sed-anaesthetic-treatment-250ml-410-p.asp
Benchtop incubator	Online Reptile Shop	https://www.onlinereptileshop.co.uk/lucky-reptile-herp-nursery-ii-incubator.html
P1000 pipette	Gilson	F144059M
P1000 pipette tips	Brand	Z740105
P200 pipette tips	Brand	Z740105
Zebrafish: strain AB	University of Portsmouth Fish Facility	N/A

## Step-by-step method details

### Habituation to new environment

**Timing: 30 min**

***Note:*** If behavior takes place in the same room as the holding/rearing facility, skip to step 2.

1. At 8:30 am, transfer the Petri dishes of 4 dpf larvae from the fish facility holding room to the experimental room and place in a benchtop incubator set at 28°C for 30-min of acclimating in the light.

***Note:*** Check the time required for your software to reach the desired temperature and allow adequate timing before experimentation.

**CRITICAL:** The experimenter can start the experiment at any time during the working day (9:00–17:00). However, all recordings must take place during the working day because of the effect of time of day on behavior (see Hillman et al.^1^). There is no requirement for each experiment to be conducted at the same time in the day because the normalization approach employed overcomes time-constraint limitations.

2. Switch on the Zantiks behavioral MWP unit(s) (or your automated tracking unit) and allow the temperature to get to 28°C, also switch on the lights in the unit (350 lx, white light).

### Plating larvae

**Timing: 8 min/plate**

This step involves the transfer of the larvae from their Petri dishes to the 48-well plate.

3. Using a standard p1000 with the tip cut approximately 2 mm from the end, transfer 225 μL of facility water and a randomly selected larva from the Petri dish into each well of a 48-well plate. Ensure the larvae are selected at random from all the Petri dishes to ensure randomization.

***Note:*** Our evidence has shown that 24, 48 and 96-well plates are all suitable for this assay.^1^ However, we recommend the 48-well plate for enhanced throughput and experimental efficiency. The volume for 24-well plates is 500 μL and for 96-well plates is 150 μL.

### Acclimation to the ZANTIKS MWP behavioral unit

**Timing: 30 min**

Here we specify the timing and criteria for the acclimation to the behavioral units.

4. Transfer the full well plate with the lid on the behavioral unit with the lights on for 30-min of untracked acclimation.

**CRITICAL:** The lid must be kept on throughout recording to ensure no evaporation of water from the wells.

**CRITICAL:** The front of the unit must be covered to prevent light from entering and impacting the behavioral responses. This can be done using the Zantiks foam covers that come with the units.

### Baseline recording and chemical exposure preparation

**Timing: 30 min**

Here we specify the timing and criteria for the baseline recording and for preparing the drug exposure solutions.

5. Run a baseline recording using the ZANSCRIPT tracking script (OSF), this includes the 5-min light to 5-min dark phase transitions repeated three times for a total of 30-min.

**CRITICAL:** If you are running more than one plate then leave 5-min in between baseline recordings to give you time for exposures.

**Figure 2:**
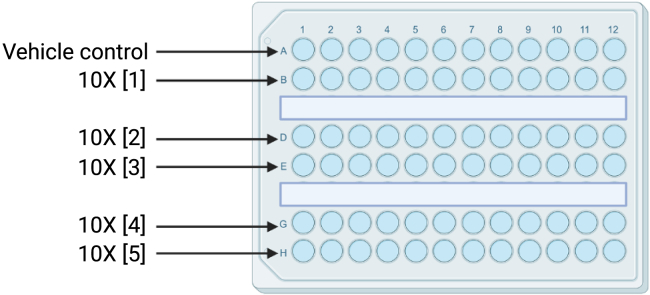
Example of a 96-well plate set up with increasing 10X concentration from A to H The vehicle control should also be a 10X concentration (e.g., for a final concentration of 0.5% DMSO, 5.0% DMSO should be made up in the 96 well plate). Rows C and F are kept empty.

6. Immediately after starting the baseline recording, prepare your 10X solutions for drug exposure in a 96-well plate (see Figure 2; Table 1).

**Table 1:** Example of the pharmacological calculations required for dosing of 4 dpf larvae

Desired exposure concentration (μM)	10X concentration (μM)	Required stock concentration (mM)	Volumes
0	N/A	N/A	25 μL of DMSO + 475 μL embryo medium
0.5	5	0.1	25 μL of 0.1 mM stock + 475 μL embryo medium
5	50	1	2.5 μL of 10 mM stock + 22.5 μL DMSO + 475 μL embryo medium
50	500	10	25 μL of 10 mM stock + 475 μL embryo medium
500	5,000	100	2.5 μL of 1M stock + 22.5 μL DMSO + 475 μL embryo medium
5,000	50,000	1,000	25 μL of 1M stock + 475 μL embryo medium

This ensures a final volume of 500 μL which is sufficient for dosing *n* = 16 larvae with a final vehicle concentration of 0.5% for all exposure concentrations.

***Note:*** We add 25 μL of the 10X concentration to the larvae; therefore, we ensure a volume of at least 30 μL in the well of the 96 well plate, to minimize pipetting errors. All drug exposures are performed using the same vehicle to ensure consistency throughout. We use dimethyl sulfoxide (DMSO) at a final exposure concentration of 0.5% (v/v)^2^. Table 1 provides a detailed summary of the requirements for a final 10X exposure volume of 500 μL, which is enough to exposure 16 larvae (2 well plates).

### Dosing the larvae and recording exposure response

**Timing: 2.5 h**

The next stage of the protocol describes the steps required for exposure and recording of behavioral responses.

***Note:*** Keep a steady hand whilst moving the plates.

**Figure 3:**
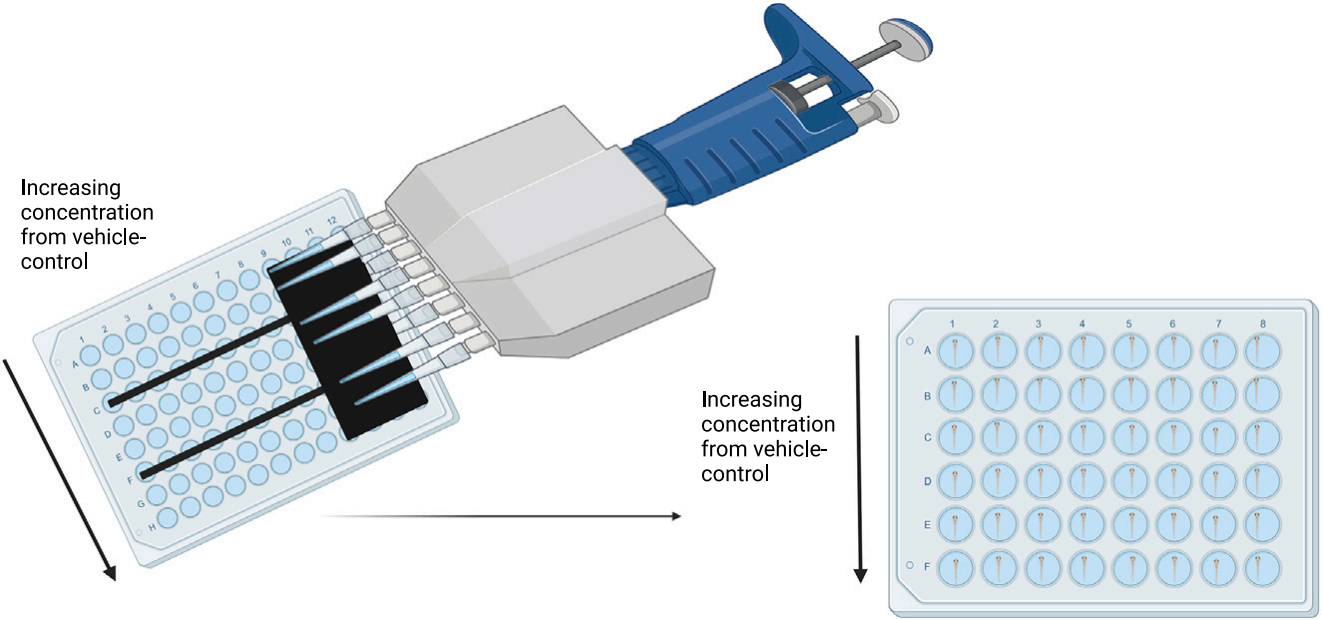
Schematic of the dosing process using an 8 channel multi-channel pipette with prepared drug solution from a 96-well plate

***Note:*** The pipette tips are removed from the third and sixth channels of an 8-channel pipette to match with the 96-well plate set-up and the 48-well plate.

7. Immediately after the baseline recording finishes remove the plate from the behavioral unit in preparation of exposures.
8. Using a multi-channel pipette, add 25 μL of the 10X dilutions from the 96-well plate to the corresponding well of the 48-well plate (See Figure 3 for reference).

**CRITICAL:** This step must be completed as quickly as possible to ensure all fish are exposed at the same time for accurate behavioral recording.

9. Once all the wells have been exposed, return the well plate with the lid on into the behavioral unit and restart the light/dark tracking script for a 0–30-min post-exposure recording.

***Optional:*** The protocol is flexible and therefore, if required by the researcher, the assay can be extended for prolonged exposure (e.g., following the 0–30 min exposure leave the fish in the unit with the lights on for an untracked break period, the script can then be re-run for a 60–90 min exposure recording).

**CRITICAL:** If you are running an extra 60–90-min recording the light untracked period is essential to prevent habituation to the light/dark transitions (see Hillman et al.^1^).

***Note:*** This process can be continued for as long as the experimenter requires.

10. Once complete, remove the fish and dispose of as appropriate.

***Note:*** We use 2-phenoxyethanol (Aqua-Sed Anesthetic by Vertak, Winchester, UK). After exposure, ensure death by maceration.

11. Download all the datafiles generated by the tracking script for analysis.

### Analytical process

**Timing: 1 h**

Here we will detail the steps required to perform the analysis using the custom R script.

***Note:*** In the example we have shown *n* = 16, which is what we recommend as the sample size for a preliminary run.

12. Save and rename the baseline recordings as “baseline_x.xlsx” where x is an increasing number from 1 corresponding to how many baseline runs were performed.
13. Create a new Microsoft Excel document for the exposed analysis with the concentration in row 1 and the 1800 s movement data in the following rows (see the example Excel data in the OSF linked to this protocol: OSF).
14. Open a new R project and upload the custom R script (OSF).
15. Change the group sizes to the sample sizes used within your experiment in lines 38, 299, 532, 715, 736, 835.

> # Calculate movement per minute for each fish (each column)

> num_fish <- ncol(data_subset)

> group_sizes <- c(**16, 16, 16, 16, 16, 16**) #Group sizes INPUT YOUR SAMPLE SIZES FROM EXPERIMENT HERE

16. Change the file path to your own file path where the saved datafiles are in lines 137 and 483.

> # Process dataset

> dataset_1_data <- process_dataset(“**INPUT YOUR FILE PATH HERE FOR THE EXPOSED DATASET**”)

17. Change the file path to your own file path where the saved baseline files are for lines 245, 248, 478, 481.

> # Process baseline_1 dataset

> baseline_1_data <- process_baseline(“baseline_1”, “**INPUT YOUR FILE PATH HERE FOR THE EXPOSED DATASET**.xlsx”)

> # Process baseline_2 dataset

> baseline_2_data <- process_baseline(“baseline_2”, “**INPUT YOUR FILE PATH HERE FOR THE EXPOSED DATASET**.xlsx”)

18. Change the lines for calculating the mean and standard error of the mean (SEM) for producing the line graph in lines 359–364 and 376–381.

> # Initialize an empty dataframe to store the means

> mean_df <- data.frame(Time = 1:30)

> # Calculate the means for each group, ignoring NA values

> mean_df$Group_1 <- apply(dataset_normalized_minute_analysis[**,1:16**],1,mean, na.rm = TRUE)

> mean_df$Group_2 <- apply(dataset_normalized_minute_analysis[**,17:32**],1,mean, na.rm = TRUE)

> mean_df$Group_3 <- apply(dataset_normalized_minute_analysis[**,33:48**],1,mean, na.rm = TRUE)

> mean_df$Group_4 <- apply(dataset_normalized_minute_analysis[**,49:64**],1,mean, na.rm = TRUE)

> mean_df$Group_5 <- apply(dataset_normalized_minute_analysis[**,65:80**],1,mean, na.rm = TRUE)

> mean_df$Group_6 <- apply(dataset_normalized_minute_analysis[**,81:96**],1,mean, na.rm = TRUE)

> # Print the resulting dataframe

> print(mean_df)

>##############################

>#Initialize an empty dataframe to store the SEM values

>sem_df <- data.frame(Time = 1:30)

># Calculate the SEM for each group, ignoring NA values

>sem_df$Group_1 <- apply(dataset_normalized_minute_analysis[**, 1:16**], 1, function(x) sd(x, na.rm = TRUE / sqrt(sum(!is.na(x))))

>sem_df$Group_2 <- apply(dataset_normalized_minute_analysis[**, 17:32**], 1, function(x) sd(x, na.rm = TRUE / sqrt(sum(!is.na(x))))

>sem_df$Group_3 <- apply(dataset_normalized_minute_analysis[**, 33:48**], 1, function(x) sd(x, na.rm = TRUE / sqrt(sum(!is.na(x))))

>sem_df$Group_4 <- apply(dataset_normalized_minute_analysis[**, 49:64**], 1, function(x) sd(x, na.rm = TRUE / sqrt(sum(!is.na(x))))

>sem_df$Group_5 <- apply(dataset_normalized_minute_analysis[**, 65:80**], 1, function(x) sd(x, na.rm = TRUE / sqrt(sum(!is.na(x))))

>sem_df$Group_6 <- apply(dataset_normalized_minute_analysis[**, 81:96**], 1, function(x) sd(x, na.rm = TRUE / sqrt(sum(!is.na(x))))

19. Change the labels for the line graph to match your tested concentrations/exposure solutions (line 456).

>scale_color_manual(values = group_colors, labels = c(“**Change to match your concentrations**”)) +

>theme_minimal()+

>theme(…

20. Change the lines corresponding to the sample size for vehicle-controls in the swim phenotypes analysis (lines 672 and 675).

>#Select rows corresponding to control fish

>control_fish_light_raw <- light_phase_results_raw[**1:16,** ]

>#Select rows corresponding to control fish

>control_fish_dark_raw <- dark_phase_results_raw[**1:16,** ]

21. Change to match your concentrations/exposure solutions and the sample sizes for the swim phenotype analysis (lines 712 and 715).

>#Define group names

>group_names <- c(“**Change to your concentrations**”)

>#Create a new column indicating the group for each row

>group_column <- rep(group_names, c(**16, 16, 16, 16, 16, 16**))

22. Prepare to generate the heat maps by changing the group sizes and concentration labels accordingly in lines 736, 740, 748, 764, 835, 862.

>#Group sizes as per your requirement

>group_sizes <- c(**16, 16, 16, 16, 16, 16**)

>Exclude the control group

>dark_phase_results_log10 <- dark_phase_results_log10 %>% >fliter(Group != “**control**”)

>#Calculate the average response for each concentration

>average_responses_df <- dark_phase_results_log10 %>% >group_by(Group) %>%

>summarise_all(mean, na.rm = TRUE)

>#Define the desired order

>desired_order <- c(“**Change to your concentrations**”)

…

>labRow = c(“**Change to your concentrations**”)

23. To create the radar charts the concentrations must be changed and you must manually input your data from the files “average_responses_df” and “average_responses_df_light” in lines 928–33, 937, 972–78, 980 (see the example R code in OSF for reference).

>#Sample data

>data <- data.frame(

>Group = c(“**Change to your concentrations**”),

>total_movements = c(**change to the data for dark total movements**),

>zero_movements = c(**change to the data for dark zero movements**),

>darting_movements = c(**change to the data for dark darting movements**),

>steady_swim_movements = c(**change to the data for dark steady swim**),

>slow_swim_movements =c(**change to the data for dark slow swim**)

>)

>#Reorder the levels of the Group factor

>data$Group <- factor(data$Group, levels = c(“**Change to your concentrations**”))

24. To prepare the data for statistical analysis the dataset must be restructured, change this section depending on your sample sizes. Lines 1033–1045.

>#Determine the group for each fish

>if(i <= **16**) {

>fish_group <- rep(1, num_timepoints)

> } else if (i <= **32**) {

>fish_group <- rep(2, num_timepoints)

> } else if (i <= **48**) {

>fish_group <- rep(3, num_timepoints)

> } else if (i <= **64**) {

>fish_group <- rep(4, num_timepoints)

> } else if (i <= **80**) {

>fish_group <- rep(5, num_timepoints)

> } else {

>fish_group <- rep(6, num_timepoints)

> }

## Expected outcomes

**Figure 4:**
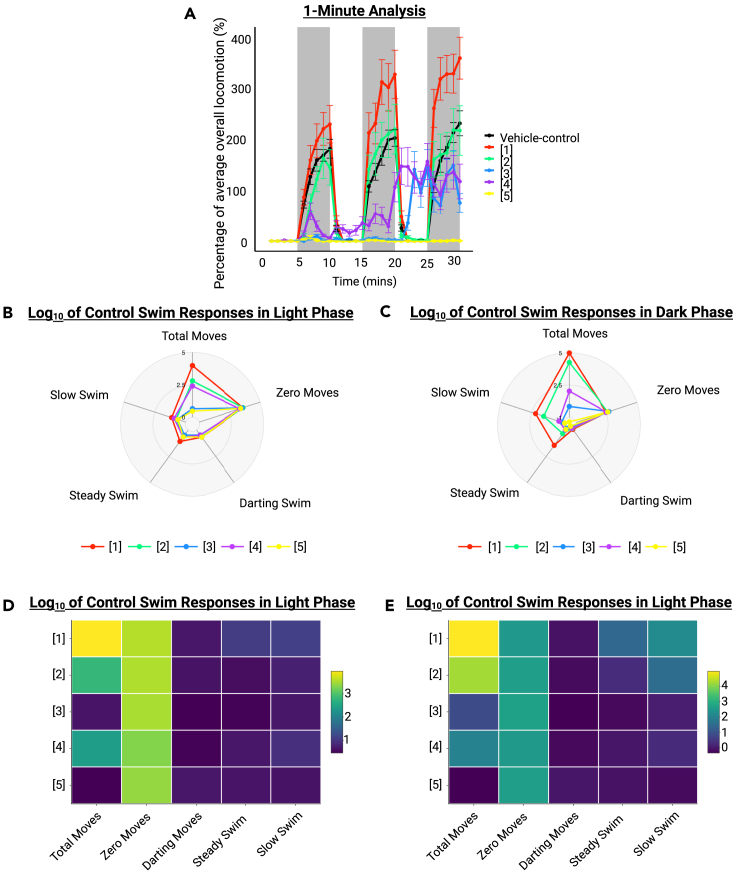
The expected figures that can be produced using the R script This demonstrates an example of the expected line graph (A), radar charts (B and C), and heat maps (D and E) that can be produced with the data generated from the protocol. Data are plotted as mean ± SEM. Scale bars in (D) and (E) represent Log10 of control swim responses in light and dark phases, respectively.

The protocol will produce at least one baseline and one 0–30 min post-exposure recording with distance traveled in mm/s/fish as the output over an 1800 s period. These files can then be used in the custom R script linked to this protocol (OSF) to produce a range of findings. Firstly, the overall locomotion for each exposure concentration over a 30-min period will be determined and visualized using a line graph (Figure 4A). Next, the script will compute the different swim phenotypes (total movements, zero movements, darting movements, steady swim movements and slow swim movements) that the fish display, represented as a percentage of vehicle-exposed control fish responses. These findings are then visualized using radar charts (Figures 4B and 4C) and/or heat maps (Figures 4D and 4E). For further information regarding the swim phenotypes and their interpretation, see Hillman et al.^1^

**Figure 5:**
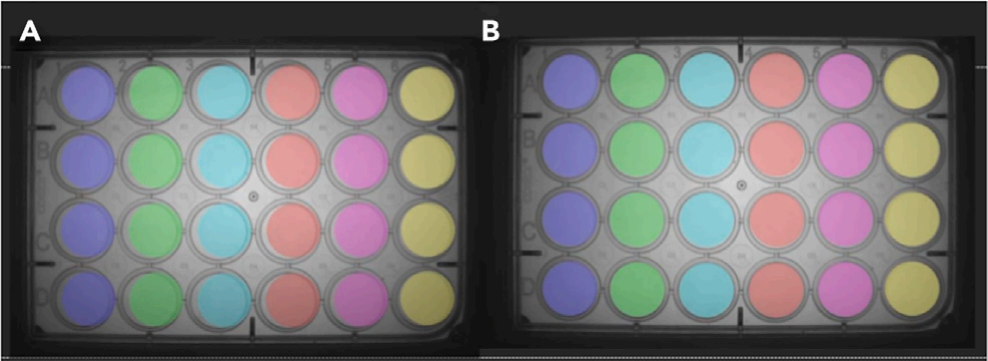
Example of asset alignment (A) represents a poorly aligned asset and (B) a perfectly aligned asset for a 24-well plate. The images are obtained from https://zantiks.com/calibration-and-asset-building-mwp.

## Quantification and statistical analysis

Within the custom R script, the data is normalized by calculating the percentage movement/min/fish compared to the overall baseline locomotion. This step is essential to ensure reproducible and robust responses (see Hillman et al.^1^). Tracking errors are common and are identified and eliminated within the script using the common method for outlier removal, median absolute deviation.^3^

The script also computes the observed power and sample size required for the data. A preliminary behavioral experiment must be conducted with a sample size of *n* = 16 per concentration. The R script then computes the observed power and required sample size. If the observed power and required sample size are sufficient (> 0.8 and < 16, respectively) then the sample size can remain as *n* = 16.

At this stage, it is up to the researcher as to what statistical analyses they wish to be conducted. We recommend that the researcher tests the normality and distributions of the data prior to running any statistical analyses. In our lab, we tend to run linear mixed model analyses to consider variability between the fish as a random effect (see Hillman et al.^1^ for further information).

## Limitations

Although an extremely reliable and effective behavioral protocol, we acknowledge potential limitations. Unfortunately, working with live animals can be temperamental and the larvae may be unresponsive to the light/dark transitions and/or the chemical exposures for unknown reasons. This is something that cannot be predicted and therefore the researcher must be aware and monitor the live responses to ensure predictable hypolocomotion in the light and hyperlocomotion in the dark phases are observed (this can be achieved by monitoring the live video of the baseline recording). Additionally, we appreciate that the custom behavioral tracking code is specific for Zantiks MWP units, which not every laboratory will have. Therefore, there may be an additional level of preparation required by the researcher to ensure their tracking software is running as predicted. This may also mean the custom R code needs adapting to match with the individual’s unique output rather than matching to the Zantiks output.

## Troubleshooting

### Problem 1

Issues when tracking larvae (step 5).

### Potential solution

Tracking errors are typically seen with poor tracking settings on the MWP unit. The script we have provided should ensure this doesn’t occur. However, there may be an issue with the asset set up on your unit. Each unit should have a uniquely set up asset matching to the 48-well plate you are using (Figure 5). Zantiks provide a step-by-step guide for adding your own assets to the MWP unit (https://zantiks.com/manuals/asset-building-in-the-mwp).

### Problem 2

Limited baseline locomotion.

### Potential solution

If the fish are unresponsive to the light/dark responses in the baseline, end the recording and start again with a new batch of fish. If with a new batch of fish the issue persists, ensure that the temperature is correct in the units and that there are no extensive vibrations or sounds near the units as this can impact the behavioral responses. If the issue persists further, it may be that the fish are from poor breeders and therefore we would recommend starting again with freshly bred embryos.

### Problem 3

Poor production of embryos during breeding.

### Potential solution

Occasionally the fish do not produce sufficient embryos for no specific reason. To monitor breeding and fecundity, keep sufficient records of breeding and ensure at least a two-week break between breeding to ensure healthy embryos and prevent overbreeding the adult fish.

## Resource availability

### Lead contact

Further information and requests for resources should be directed to and will be fulfilled by the lead contact, Matthew O. Parker (matthew.parker@surrey.ac.uk).

### Technical contact

All technical questions regarding completion of the protocol should be directed to and will be fulfilled by the technical contact, Courtney Hillman (c.hillman@surey.ac.uk).

### Materials availability

This study did not generate any new unique reagents.

### Data and code availability

• Original/source data used in the live example and for data shared here are available at OSF.
• The custom R code is available at OSF.
• The ZANSCRIPT tracking script is available here OSF.

## Acknowledgments

We would like to thank Charlotte White and Laura Palmer at the University of Portsmouth for their continued support and help in the breeding and care of the fish. We would also like to thank Bill, Shanelle, and Jenny at Zantiks for their support and assistance. C.H. is funded by the Defense Science and Technology Laboratory (DSTL) and J.K. and M.O.P. are funded by NC3Rs (NC/W00092X/1).

## Author contributions

Conceptualization, C.H., J.K., and M.O.P.; investigation, C.H.; writing – original draft, C.H.; writing – review and editing, C.H., J.K., and M.O.P.; funding acquisition, J.K. and M.O.P.; supervision, J.K. and M.O.P.

## Declaration of interests

The authors declare no competing interests.
